# Effects of Psychosomatic Mutual Aid Treatment on Anxiety and Depression in Turner Syndrome

**DOI:** 10.3389/fpsyt.2021.644147

**Published:** 2021-05-10

**Authors:** Mudan Ye, Huijia Lin, Gendie E. Lash, Lianxiong Yuan, Li Li

**Affiliations:** ^1^Department of Obstetrics and Gynecology, Guangzhou Women and Children's Medical Center, Guangzhou Medical University, Guangzhou, China; ^2^Department of Guangzhou Institute of Pediatrics, Guangzhou Women and Children's Medical Center, Guangzhou Medical University, Guangzhou, China; ^3^Department of Biostatistics, Sun Yat-sen University, Guangzhou, China

**Keywords:** turner syndrome, psychosomatic mutual aid treatment, depression, anxiety, hormone replacement therapy

## Abstract

**Background:** Turner syndrome (TS) affects approximately one out of 2,500 females. Previous research indicates that girls with TS experience psychosocial impairment in addition to their physical health issues. However, there is no current data demonstrating whether reducing the clinical symptoms of girls or women with TS through hormone replacement therapy (HRT) combined with psychological interventions, referred to as psychosomatic mutual aid treatment (PMAT), improves physical and psychological self-identification, so that psychological problems such as anxiety, depression, low self-esteem, social loneliness, and psychological resilience are improved. Therefore, the objective of this research was to assess the efficacy of PMAT on anxiety and depression in girls and women with TS.

**Methods:** Twenty-six girls and women with TS aged 11–29 years (17.5 ± 4.2 years) were recruited. Anxiety and depression were assessed using Hamilton Anxiety Rating Scale (HAMA) and Zung Self-Rating Depression Scale (SDS) questionnaires, respectively. The 26 TS patients were surveyed for anxiety and depression before the beginning of PMAT and again in January 2020. In addition, 20 healthy volunteer women aged 16–39 years (23.1 ± 5.7 years) were selected as the control group and filled in the questionnaire.

**Results:** Pre-therapy (pre-HRT and Pre-PMAT) there were significant differences between the TS patients (*n* = 26) and healthy controls (*n* = 20). In particular, the TS patients had higher anxiety status (*P* = 0.04) and severity (*P* = 0.03) (HAMA score), as well as depression status (*P* = 0.002) and severity (*P* < 0.001) (SDS score). Post-therapy there was no longer any difference in depression scores, but TS patients still had higher levels of anxiety post-therapy compared with healthy control women (psychic symptoms score, *P* = 0.03; anxiety status score, *P* = 0.04; anxiety severity score, *P* = 0.04). In the TS patients, there was an improvement in depression scores (SDS score *P* < 0.001; depression severity score, *P* = 0.005) after therapy but no change in levels of anxiety.

**Conclusions:** PMAT significantly improves depression status, but not anxiety, in girls and women with TS.

**Clinical Trial Registration:** http://www.chictr.org.cn/showproj.aspx?proj=124736, identifier: ChiCTR2100045230.

## Introduction

TS is one of the most common genetic conditions caused by the complete or partial deletion of the second sex chromosome. The ovaries of people with TS are replaced by strip-shaped fibrous tissue, and make insufficient estrogen resulting in hypogonadism, primary amenorrhea, short stature, physical malformations, infertility, metabolic disorders, an increased risk of autoimmune disease, as well as other medical conditions such as cardiovascular disease. In addition, it can also be accompanied by a series of endocrine abnormalities such as glucose metabolism disorders and thyroid diseases. The incidence of TS is about 1 per 2,500 female births and is the only monomeric syndrome of which humans survive (1–3). In addition to these physical health concerns, previous research indicates that people with TS experience psychosocial impairment (4). Wolstencroft and Skuse (5) demonstrated that girls with TS experience social interaction challenges throughout their life, in particular in relationships and developing and maintaining friendship, which is associated with impaired social-cognitive processing and executive function deficit. Psychological intervention is essential for the treatment of psychological disorders. Through guidance and instruction, levels of understanding and communication can be improved, ultimately helping girls with TS to obtain family and social understanding and support (6).

Traditional psychological interventions need to be performed in specific locations and environments, with treatment often being expensive, typically with long waiting times and restricted access. Long distance medicine has become more of a reality as different messenger APPs have been developed. The use of long distance medicine for psychological therapy offers a number of advantages over traditional psychological interventions, including cost savings, increased patient adherence and acceptance. A meta-analysis review revealed that internet-delivered cognitive behavioral therapy (iCBT) and face-to-face treatment had similar efficacy for treatment of psychiatric and somatic disorders (7). Meta-analysis revealed that therapist-supported iCBT significantly improved stress, anxiety, and depressive symptoms among post-partum women (8). Therefore, it should be possible to adopt an internet and mobile phone (WeChat, a mobile-phone instant messaging social networking app widely used in China) approach along with guided face-to-face treatment for psychological intervention in girls with TS.

In addition, girls with TS have also been reported to have lower self-esteem, due to primary amenorrhea, short stature, physical malformation, infertility, and other physiological and pathological characteristics (9, 10). Studies have shown that anxiety and depression disorders affect not only psychological health but also physiological function and well-being (10). HRT is effective in alleviating many physiological symptoms for TS, in turn strengthening self-esteem and improving the psychosocial issues often experienced by girls with TS (10). For girls with TS, theoretically HRT can improve psychological disorders as a side effect of improving physical characteristics, but most of these changes are small, and psychological disorders are also closely related to personal fitness, psychological environment, family, and social environment. Therefore, it is likely that HRT alone will have low efficacy in improving psychological disorders in girls with TS. However, HRT combined with psychological intervention (psychosomatic mutual aid treatment, PMAT) may be effective in helping girls with TS improve their mental health status. Therefore, the aim of the current study was to assess the efficacy of PMAT on anxiety and depression in girls with TS.

## Methods

### Ethical Approval

The study protocol was approved by the ethics committee of Guangzhou Women and Children's Medical Center (no: 2016042019). It was conducted in accordance with the principles of the Second Revision of the Declaration of Helsinki. All participants enrolled or the parents of those girls with a chronological age below 18 years signed informed consent forms.

### Questionnaires

Anxiety severity was assessed by using the Hamilton Anxiety Rating Scale (HAMA) (11) and depression severity was evaluated with the use of the Zung Self-Rating Depression Scale (SDS) (12). These scales are validated for the assessment of depression and anxiety (13, 14).

**Figure 1:**
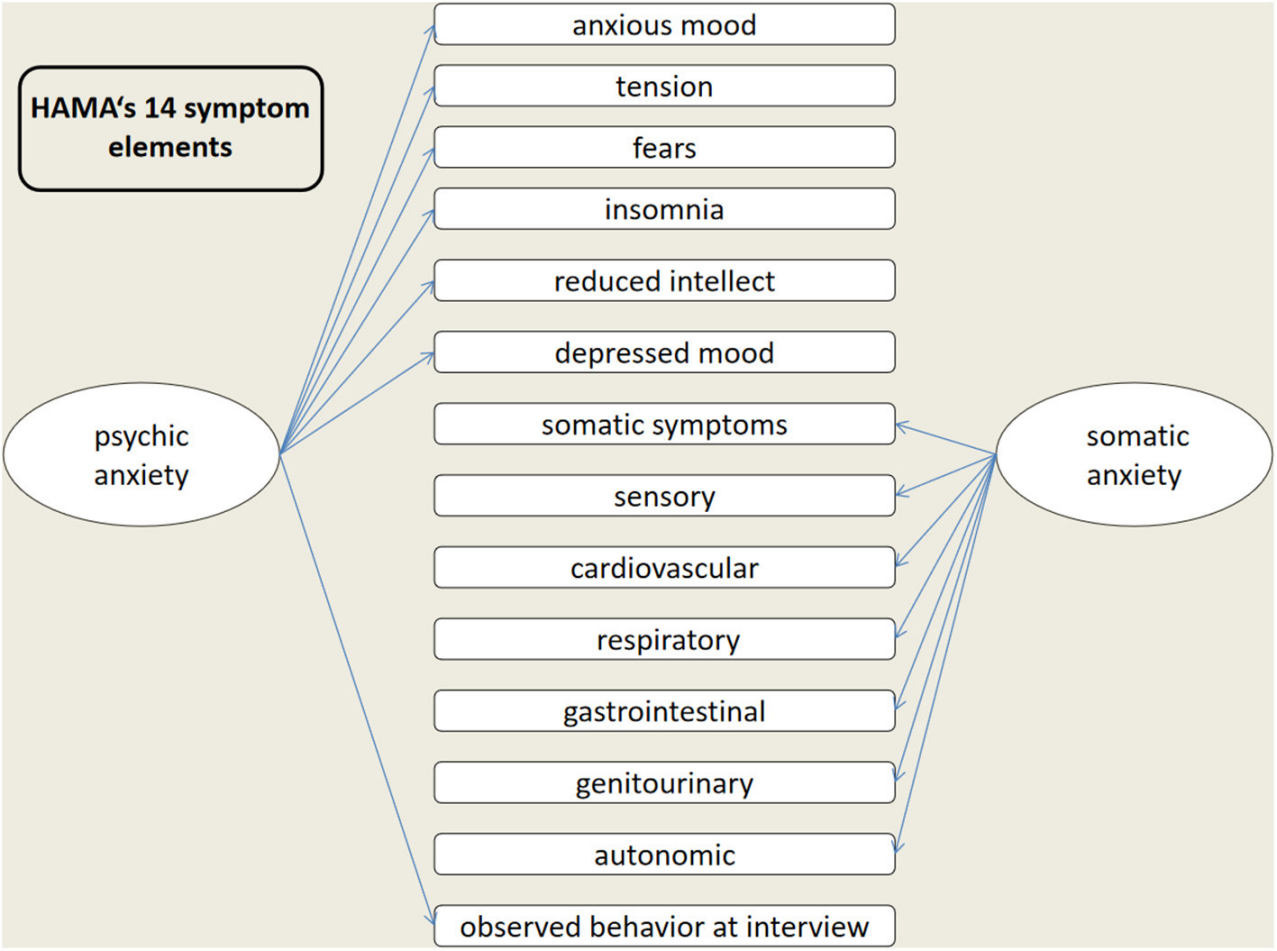
HAMA's 14 symptom elements and two structural factors.

HAMA (11) consists of 14 items and is one of the most widely used scales in psychiatry by doctors, mainly used to assess the severity of anxiety symptoms in neurosis and other patients, which are individually scored on a scale of 0–4, ranging from “asymptomatic” to “extremely severe,” resulting in a total score of 0–56 and classified as: mild anxiety, 8–14; moderate, 15–23; severe, ≥24 (scores ≤7 were considered to represent no/minimal anxiety). The HAMA scale is divided into two structural factors: psychic anxiety and somatic anxiety (Figure 1).

The SDS questionnaire for depression (12) consists of 20 questions, with 10 positively worded and 10 negatively worded items. Participants were asked to rate these items using a 1–4 point scale, ranging from “never or rarely” to “most of the time.” The total score for the 20 questions was multiplied by 1.25, with the integer score as a standard score and classified as: 25–49, no depression; 50–59, light depression; 60–69, moderate depression; 70–100, severe depression.

### Participants and Setting

Participants were recruited from 2014 to 2018 through the Department of Gynecology Endocrinology at the Guangzhou Women and Children's Medical Center, Guangzhou Medical University. After a pilot study tested the feasibility of the use of these questionnaires, the formal survey was conducted. All questionnaires were independently completed by patients before the commencement of PMAT and again in January 2020, which included basic personal information, medical history, family conditions, living habits, education and emotional status. Patients were enrolled if the following inclusion criteria were met: (1) TS was established according to cytogenetic analysis performed on peripheral blood lymphocytes; (2) Patients who had independent reading, comprehension and writing abilities; (3) Did not participate in other surveys with similar study aims. Patients who had serious physical complications, mental disorders, and consciousness disorders were excluded from the study. Finally, based on these criteria 26 women with TS aged 11–29 years (17.5 ± 4.2 years) were invited to join the WeChat group for subsequent psychological intervention and follow-up. Among them, 2 patients (8.7%) were in the sixth grade of elementary school, 7 patients (26.9%) were junior high school students, 8 patients (30.8%) were high school students, 6 patients (23.1%) were college students, and 3 patients (11.5%) were already working. The participants were treated with HRT by the same doctors in the Departments of Gynecology Endocrinology at the Guangzhou Women and Children's Medical Center, Guangzhou Medical University. In addition, 20 healthy volunteer women aged 16–39 years (23.1 ± 5.7 years) were selected as the control group and filled in the questionnaires.

All healthy volunteer control women were in good overall medical health. None of the study participants had evidence of current or past major neurological and psychiatric problems. All women with TS were diagnosed based on karyotype of one X chromosome and complete or partial absence of the second sex chromosome, associated with one or more typical clinical manifestations of TS (15). To reduce bias caused by anxiety and depression related factors including age, ethnicity, and ovarian function status, we performed this study among girls and women with TS with primary amenorrhea from China. HRT and PMAT (as described below) were started at the same time for those patients included in the current study. Participants did not seek any other psychological treatment for the duration of the study.

### Psychosomatic Mutual Aid Treatment (PMAT)

Patients younger than 15 years of age were started with low-dose estradiol (E2) administered systemically (0.5 mg daily for the first 6 months, continued with 1 mg daily for another 6 months; Progynova) and progesterone treatment began when breakthrough bleeding occurred or after 2 years of estrogen treatment. Patients over 15 years old received cyclic HRT (17β-estradiol 2 mg/d for 28 days, adding dydrogesterone 10 mg/day for 14 days on day 14; Fenmatong) (15).

The psychological intervention (cognitive behavioral therapy, interpersonal therapy, psychoeducational therapy) was started at the same time as HRT. Individual treatment plans were established based on the initial questionnaire results and discussions with the patient. Therapy sessions included interactive treatments conducted every week for 30 min on WeChat (common social media messaging app, similar to WhatsApp or Messenger) by text or video messaging, and guided face-to-face sessions every 3–6 months. Cognitive behavioral therapy allows the patient to build trust and maintain a sense of control over the chosen steps of the therapy process. The treating physicians were able to listen patiently to the patient's knowledge and feelings regarding her TS, thereby enabling the delivery of culturally sensitive, and individual patient-centered education regarding their symptoms of depression and anxiety. Interpersonal therapy focused on role transition, either in terms of life changes or in terms of a therapeutic role transition. Therapeutic role transition means that the patient recognizes that being sad is not a part of her personality but rather a temporary state or role, using role-play if indicated, and encouraging the patient to communicate and express their feelings. The interventions generally aim to enable the patient to build a social network by forming and maintaining close and trusting relationships. The psychoeducational therapy involved both parents and patients in lectures and symposiums that were held once a year to explain disease-related knowledge and encourage patients to bravely express their inner thoughts and emotions, vent negative emotions in the right way, and actively cooperate with doctors for treatment. Family members were encouraged to give patients sufficient care and understanding to help enrich the girls' daily lives.

### Statistical Analysis

Statistical analysis was performed using SPSS 22.0 software (Statistical Package of Social Sciences, Chicago, IL, USA). Normally distributed continuous variables are shown as mean ± standard deviation, non-normal continuous variables are shown as median (25th−75th quartiles), and categorized variables are expressed as count (percentage). Comparison between the two groups was determined by Student *t*-test, independent sample rank sum test, paired sample rank sum test or Chi-square test as appropriate. Statistically significant levels reported are two-sided and a *P* < 0.05 was considered significant.

In this study, the anxiety and depression scores are not normally distributed, therefore are shown as median (25th−75th quantiles), and the paired rank test or independent sample rank sum test were used for comparison. In addition, the total score is divided according to severity score and Rank-sum test used for comparison between groups, if the results of anxiety severity and depression severity had only 2 categories, then the chi-square test was used for comparison. If one of the groups has only 1 category, no comparison is possible.

## Results

Twenty-six women with TS aged 11–29 years (17.5 ± 4.2 years) completed the HAMA and SDS questionnaires at the beginning of PMAT and again in January 2020 (average treatment time 5.5 ± 3.8 years). Twenty healthy volunteer women completed the HAMA and SDS questionnaires in January 2020. All of the participants answered the questionnaires in an independent, quiet environment and took longer than 20 min.

At baseline (pre-therapy) there were significant differences between the TS patients (*n* = 26) and healthy controls (*n* = 20; Table 1). In particular, the TS patients had higher anxiety status (*P* = 0.04) and severity (*P* = 0.03) (HAMA score), as well as depression status (*P* = 0.002) and severity (*P* < 0.001) (SDS score) (Table 1). Post-therapy there was no longer any difference in depression scores, but TS patients still had higher levels of anxiety post-therapy compared with healthy control women (psychic symptoms score, *P* = 0.03; anxiety status score, *P* = 0.04; anxiety severity score, *P* = 0.04; Table 2). In the TS patients, there was a statistically significant reduction in depression severity but not presence of depression (SDS score *P* < 0.001; depression severity score, *P* = 0.005) after therapy but no change in levels of anxiety (Table 3).

**Table 1:** Anxiety and depression in pre-therapy TS patients and healthy control women.

**Items**	**Pre-therapy (*N* = 26)**	**Healthy control women (*N* = 20)**	***P***
**HAMA score**	8 (3–15)	4.5 (2.25–6.75)	1.1
Psychic symptoms score	5 (2.75–9)	3 (2–6.25)	0.1
Somatic symptoms score	2.5 (0–7.25)	0.5 (0–2)	0.1
**Anxiety status by HAMA score**			**0.037**
No anxiety (HAMA score ≤7, *n*/%)	13 (50.00%)	16 (80.00%)	
Anxiety (HAMA score >7, *n*/%)	13 (50.00%)	4 (20.00%)	
**Anxiety severity by HAMA score**			**0.025**
No/minimal anxiety (HAMA score ≤7, *n*/%)	13 (50.00%)	16 (80.00%)	
Mild anxiety (HAMA score 8–14, *n*/%)	6 (23.08%)	3 (15.00%)	
Moderate (HAMA score 15–23, *n*/%)	6 (23.08%)	1 (5.00%)	
Severe (HAMA score ≥24, *n*/%)	1 (3.84%)	0 (0.0%)	
**SDS score**	60 (50.75–63)	44.38 (35.63–55.94)	**0.004**
**Depression status by SDS score**			**0.002**
No depression (SDS score <50, *n*/%)	5 (19.2%)	13 (73.1%)	
Depression (SDS score >50, *n*/%)	21 (80.8%)	7 (26.9%)	
**Depression severity by SDS score**			**<0.001**
No (SDS score <50, *n*/%)	5 (19.2%)	13 (73.1%)	
Light (SDS score 50–59, *n*/%)	7 (26.9%)	7 (26.9%)	
Moderate (SDS score 60–69, *n*/%)	14 (53.8%)	0 (0.0%)	
Severe (SDS score 70–100, *n*/%)	0 (0.0%)	0 (0.0%)	

*Data are presented as count (percentage) or median (25th−75th quartiles). Comparison was determined by student t-test, Chi-square test or Wilcoxon rank sum test. P < 0.05 was considered significant (in bold). HAMA, the Hamilton Anxiety Rating Scale; SDS, the Zung Self-Rating Depression Scale*.

**Table 2:** Anxiety and depression in post-therapy TS patients and healthy control women.

**Items**	**Post-therapy (*N* = 26)**	**Healthy control women (*N* = 20)**	***P***
**HAMA score**	7.5 (3–14)	4.5 (2.25–6.75)	0.07
Psychic symptoms score	6 (3–10)	3 (2–6.25)	**0.03**
Somatic symptoms score	2 (0–4)	0.5 (0–2)	0.2
**Anxiety status by HAMA score**			**0.037**
No anxiety (HAMA score ≤7, *n*/%)	13 (50.00%)	16 (80.00%)	
Anxiety (HAMA score >7, *n*/%)	13 (50.00%)	4 (20.00%)	
**Anxiety severity by HAMA score**			**0.04**
No/minimal anxiety (HAMA score ≤7, *n*/%)	13 (50.00%)	16 (80.00%)	
Mild anxiety (HAMA score 8–14, *n*/%)	8 (30.77%)	3 (15.00%)	
Moderate (HAMA score 15–23, *n*/%)	5 (19.23%)	1 (5.00%)	
Severe (HAMA score ≥24, *n*/%)	0 (0.00%)	0 (0.0%)	
**SDS score**	51 (40.5–56)	44.38 (35.63–55.94)	0.3
**Depression status by SDS score**			0.1
No depression (SDS score <50, *n*/%)	11 (42.3%)	13 (73.1%)	
Depression (SDS score >50, *n*/%)	25 (57.7%)	7 (26.9%)	
**Depression severity by SDS score**			0.1
No (SDS score <50, *n*/%)	11 (42.3%)	13 (73.1%)	
Light (SDS score 50–59, *n*/%)	12 (46.2%)	7 (26.9%)	
Moderate (SDS score 60–69, *n*/%)	3 (11.5%)	0 (0.0%)	
Severe (SDS score 70–100, *n*/%)	0 (0.0%)	0 (0.0%)	

*Data are presented as count (percentage) or median (25th−75th quartiles). Comparison was determined by student t-test, Chi-square test or Wilcoxon rank sum test. P < 0.05 was considered significant (in bold). HAMA, the Hamilton Anxiety Rating Scale; SDS, the Zung Self-Rating Depression Scale*.

**Table 3:** Anxiety and depression in TS patients pre- and post-therapy.

**Items**	**Pre-therapy (*N* = 26)**	**Post-therapy (*N* = 26)**	***P***
**HAMA score**	8 (3–15)	7.5 (3–14)	0.9
Psychic symptoms score	5 (2.75–9)	6 (3–10)	0.2
Somatic symptoms score	2.5 (0–7.25)	2 (0–4)	0.06
**Anxiety status by HAMA score**			1.0
No anxiety (HAMA score ≤7, *n*/%)	13 (50.00%)	13 (50.00%)	
Anxiety (HAMA score >7, *n*/%)	13 (50.00%)	13 (50.00%)	
**Anxiety severity by HAMA score**			0.629
No/Minimal anxiety (HAMA score ≤7, *n*/%)	13 (50.00%)	13 (50.00%)	
Mild anxiety (HAMA score 8–14, *n*/%)	6 (23.08%)	8 (30.77%)	
Moderate (HAMA score 15–23, *n*/%)	6 (23.08%)	5 (19.23%)	
Severe (HAMA score ≥24, *n*/%)	1 (3.84%)	0 (0.00%)	
**SDS score**	60 (50.75–63)	51 (40.5–56)	**<0.001**
**Depression status by SDS score**			0.07
No depression (SDS score <50, *n*/%)	5 (19.2%)	11 (42.3%)	
Depression (SDS score >50, *n*/%)	21 (80.8%)	15 (57.7%)	
**Depression severity by SDS score**			**0.005**
No (SDS score <50, *n*/%)	5 (19.2%)	11 (42.3%)	
Light (SDS score 50–59, *n*/%)	7 (26.9%)	12 (46.2%)	
Moderate (SDS score 60–69, *n*/%)	14 (53.9%)	3 (11.5%)	
Severe (SDS score 70–100, *n*/%)	0 (0.0%)	0 (0.0%)	

*Data are presented as count (percentage) or median (25th−75th quartiles). Comparison was determined by student t-test, Chi-square test or Wilcoxon rank sum test. P < 0.05 was considered significant (in bold). HAMA, the Hamilton Anxiety Rating Scale; SDS, the Zung Self-Rating Depression Scale*.

## Discussion

To improve quality of life is one of the fundamental purposes of health care. For girls and women with TS, previous studies have shown that increasing height, inducing puberty and treatment with growth hormone are effective in improving quality of life and psychological well-being (16, 17). To the best of our knowledge this is the first study to investigate the efficacy of PMAT in TS patients in terms of anxiety and depression, and the results are optimistic.

In the current study we demonstrated that in the absence of hormone and psychological treatment the girls with TS had higher levels of depression and anxiety than controls, consistent with previous research (9, 18). As a rule, girls with TS have lower self-esteem than that of peers without TS, but there are many factors that can impair self-esteem (10, 18). One possible reason is that the patient's anxiety about gonadal dysplasia is different from their normal peers, and the anxiety of TS patients combined with abnormal lipid metabolism, cardiovascular disease, and other complications, which is manifested as somatic discomfort (somatization), along with interpersonal communication obstacles, depression, and anxiety, will gradually affect participation in social activities, exacerbate patient feelings of inferiority, cause fear of social activities, and lead to psychological disorders. Using a vulnerability and scar model Sowislo and Orth (19) demonstrated that depression and self-esteem are closely connected, the vulnerability model indicated that low self-esteem contributes to depression, and that in turn depression erodes self-esteem.

Improvement of mental health well-being in girls and women with TS depends on both physiological and psychological treatment. The current study used PMAT, which improved patients' feelings of depression, but not their anxiety levels. One reason may be because ~58% of the TS patients in this study were junior high school students and high school students during the study period, previous research suggests these patients have executive selective learning difficulties, especially in mathematical competencies (20). The combination of upcoming final exams, at the time of the follow-up questionnaires, may also have increased anxiety. A study of 724 high school students found that examinations increased anxiety and reduced self-esteem in senior high school students, especially girls (21).

TS is usually accompanied by gonadotropin hypogonadism and primary hypogonadism. About one-third of girls with TS have spontaneous menstruation, usually in patients with a mosaic karyotype (22). Most patients with TS will need HRT to induce puberty and for maintaining female secondary sex characteristics, attaining peak bone mass and normalizing uterine growth (1, 23). Studies have shown that having pubertal development at the right age has a positive effect on the psychological well-being of girls with TS (16). Puberty should be induced at a physiologically appropriate age in patients with Turner syndrome to optimize self-esteem, social adjustment, and initiation of the patient's sex life (24). In addition, the effects of life, family and social environment for mental health well-being need to be improved through psychological intervention. Chadwick et al. (6) demonstrated that generic skills-based psychological interventions improve self-esteem and reduce psychological distress in patients with TS. The USPSTF (US Preventive Services Task Force) recommends screening for depression in the general adult population in the United States (25), which is the best way of maintaining visits to primary-care providers. Regular screenings for psychological disorders in girls or women with TS are important, which will help doctors intervene in a timely manner for those patients with TS who are displaying symptoms of psychological disorders. Wustmann and Preuss (26) published a case report in a 50-year-old TS patient, suffering from depression and schizophrenia due to various complications of TS, both of which improved after medication and psychological intervention. Wang et al. (27) investigated the effect of drug therapy and psychological intervention in patients with persistent moderate to severe allergic rhinitis, showing that combination therapy was more effective than medication alone in reducing anxiety and depression in patients, and significantly improving their quality of life.

Due to the relative rarity of TS the current study has a limited sample size and larger studies are required. In addition, we report a short timeframe longitudinal study, which only shows the effect of PMAT on anxiety and depression at one (unfixed) time point, and further long-term follow-up studies and continuous dynamic observation at different times are needed to evaluate the long-term efficacy of PMAT for girls and women with TS during their lifetime. In addition, because we do not have HRT or psychological intervention groups alone we are not able to separate out the relative effects of each treatment modality. Because TS patients are diagnosed at different times, patients with a wide range of ages (11–29 years) were included in the study, which may also have affected the results as they were going through different periods of their lives. The control group was also slightly older than the sample group and did not include girls of high school age, which we think may have been a confounder to the anxiety scores in the TS group. The anxiety questionnaire used was administered by the therapist, whereas the depression questionnaire was self-administered, future studies will include a self-administered anxiety questionnaire to determine the patients' assessment of their anxiety levels. In further studies, we will group age, time of diagnosis, family situation, and academic performance in more detail to reduce the impact of these confounders on the results.

In conclusion, our findings suggest that the PMAT can significantly improve psychological disorders in girls and women with TS, particularly depression but not anxiety, benefiting their long-term quality of life.

## Data Availability Statement

The original contributions presented in the study are included in the article, further inquiries can be directed to the corresponding author.

## Ethics Statement

The studies involving human participants were reviewed and approved by Guangzhou Women and Children's Medical Center (no: 2016042019). Written informed consent to participate in this study was provided by the participants' legal guardian/next of kin.

## Author Contributions

LL conceptualized and designed the study, reviewed coordinated and supervised data collection, critically reviewed the manuscript for important intellectual content, and revised the manuscript. MY designed questionnaire, collected data, and drafted the initial manuscript. GL reviewed and revised the manuscript. LY carried out the initial analysis. HL collected data. All authors reviewed and approved the final manuscript as submitted and agree to be accountable for all aspects of the work.

## Conflict of Interest

The authors declare that the research was conducted in the absence of any commercial or financial relationships that could be construed as a potential conflict of interest.

## Abbreviations

TS: turner syndrome
HRT: hormone replacement therapy
PMAT: Psychosomatic Mutual Aid Treatment
HAMA: the Hamilton Anxiety Rating Scale
SDS: the Zung Self-Rating Depression Scale
iCBT: internet-delivered cognitive behavioral therapy
BMD: bone mineral density
MHT: menopausal hormone therapy
USPSTF: US Preventive Services Task Force.

## References

[1] Gravholt CH Backeljauw P. New international turner syndrome guideline: a multi-society feat. Eur J Endocrinol. (2017) 177:E1–2. 10.1530/EJE-17-0540 28705802

[2] Wang H Zhu H Zhu W Xu Y Wang N Han B. Bioinformatic analysis identifies potential key genes in the pathogenesis of turner syndrome. Front Endocrinol. (2020) 11:104. 10.3389/fendo.2020.00104 32210915 PMC7069359

[3] Sun L Wang Y Zhou T Zhao X Wang Y Wang G. Glucose metabolism in turner syndrome. Front Endocrinol. (2019) 10:49. 10.3389/fendo.2019.00049 PMC6374553 30792694

[4] Reis CT de Assumpção MS Guerra-Junior G de Lemos-Marini SHV. Systematic review of quality of life in turner syndrome. Qual Life Res. (2018) 27:1985–2006 10.1007/s11136-018-1810-y 29427215

[5] Wolstencroft J Skuse D. Social skills and relationships in turner syndrome. Curr Opin Psychiatry. (2019) 32:85–91. 10.1097/YCO.0000000000000472 30407217

[6] Chadwick PM Smyth A Liao L. Improving self-esteem in women diagnosed with turner syndrome: results of a pilot intervention. J Pediatr Adol Gynec. (2014) 27:129–32. 10.1016/j.jpag.2013.09.004 24656696

[7] Holst A Bjorkelund C Metsini A Madsen JH Hange D Petersson EL. Cost-effectiveness analysis of internet-mediated cognitive behavioural therapy for depression in the primary care setting: results based on a controlled trial. BMJ Open. (2018) 8:e019716. 10.1136/bmjopen-2017-019716 29903785 PMC6009451

[8] Lau Y Htun TP Wong SN Tam WSW Klainin-Yobas P. Therapist-supported internet-based cognitive behavior therapy for stress, anxiety, and depressive symptoms among postpartum women: a systematic review and meta-analysis. J Med Internet Res. (2017) 19:e138. 10.2196/jmir.6712 28455276 PMC5429436

[9] Liedmeier A Jendryczko D van der Grinten HC Rapp M Thyen U Pienkowski C. Psychosocial well-being and quality of life in women with turner syndrome. Psychoneuroendocrinology. (2019) 113:104548. 10.1016/j.psyneuen.2019.104548 31923612

[10] Schmidt PJ Cardoso GMP Ross JL Haq N Rubinow DR Bondy CA. Shyness social anxiety, and impaired self-esteem in turner syndrome and premature ovarian failure. JAMA. (2006) 295:1373–8. 10.1001/jama.295.12.1374 16551707

[11] Matza LS Morlock R Sexton C Malley K Feltner D. Identifying HAM-A cutoffs for mild, moderate, and severe generalized anxiety disorder. Int J Meth Psych Res. (2010) 19:223–32. 10.1002/mpr.323 20718076 PMC6878292

[12] Wechsler H Grosser GH Busfield JBL. The depression rating scale: a quantitative approach to the assessment of depressive symptomatology. Archiv General Psychiatry. (1963) 9:334. 14045262 10.1001/archpsyc.1963.01720160024003

[13] Gravholt CH Andersen NH Conway GS Dekkers OM Geffner ME Klein KO. Clinical practice guidelines for the care of girls and women with turner syndrome: proceedings from the 2016 Cincinnati International Turner Syndrome Meeting. Eur J Endocrinol. (2017) 177:G1–70. 10.1530/EJE-17-04301 28705803

[14] Clark DB Donovan JE. Reliability and validity of the Hamilton Anxiety Rating Scale in an adolescent sample. J Am Acad Child Psy. (1994) 33:354. 8169180 10.1097/00004583-199403000-00009

[15] Gabrys JB Peters K. Reliability discriminant and predictive validity of the Zung Self-Rating Depression Scale. Psychol Rep. (1985) 57(Suppl. 3):1091–6. 10.2466/pr0.1985.57.3f.1091 4095223

[16] Bannink EMN Raat H Mulder PGH de Muinck Keizer-Schrama SMPF. Quality of life after growth hormone therapy and induced puberty in women with turner syndrome. J Pediatr. (2006) 148:95–101. 10.1016/j.jpeds.2005.08.043 16423606

[17] Kosteria I Kanaka-Gantenbein C. Turner syndrome: transition from childhood to adolescence. Metabolism. (2018) 86:145–53. 10.1016/j.metabol.2017.12.016 29309748

[18] Kiliç BG Ergür AT Ocal G. Depression levels of anxiety and self-concept in girls with turner's syndrome. J Pediatr Endocrinol Metab. (2005) 18:1111–7. 10.1515/jpem.2005.18.11.1111 16459458

[19] Sowislo F Orth U. Does low self-esteem predict depression and anxiety? A meta-analysis of longitudinal studies. Psychol Bull. (2013) 139:213–40. 10.1037/a0028931 22730921

[20] Baker M Reiss AL. A meta-analysis of math performance in turner syndrome. Dev Med Child Neurol. (2016) 58:123–30. 10.1111/dmcn.12961 26566693 PMC4724271

[21] Da Silva Negreiros P Bolina ER Guimarães MM. Pubertal development profile in patients with turner syndrome. J Pediatr Endocrinol Metab. (2014) 27:845–9. 10.1515/jpem-2013-0256 24887955

[22] Muscogiuri G Altieri B de Angelis C Palomba S Pivonello R Colao A. Shedding new light on female fertility: the role of vitamin D. Rev Endocr Metab Disord. (2017) 18:273–83. 10.1007/s11154-017-9407-2 28102491

[23] Guo S Zhang J Li Y Ma H Chen Q Chen H. The pubertal development mode of Chinese girls with turner syndrome undergoing hormone replacement therapy. BMC Endocr. Disord. (2019) 19:72. 10.1186/s12902-019-0403-2 31296213 PMC6625027

[24] Carel J Elie C Ecosse E Tauber M Léger J Cabrol S. Self-esteem and social adjustment in young women with turner syndrome—influence of pubertal management and sexuality: population-based cohort study. J Clin Endocrinol Metab. (2006) 91:2972–9. 10.1210/jc.2005-2652 16720662

[25] Siu L Bibbins-Domingo K Grossman DC Baumann LC Davidson KW Ebell M. Screening for depression in adults: US preventive services task force recommendation statement. JAMA. (2016) 315:380–7. 10.1001/jama.2015.18392 26813211

[26] Wustmann T Preuss UW. Turner-syndrome and psychosis: a case report and brief review of the literature. Psychiatr Praxis. (2009) 36:243. 10.1055/s-2008-1067547 18802878

[27] Wang L Yang Z Kang Z Di L Tan Y Peng X. Improvement in psychological condition of patients with persistent moderate-severe allergic rhinitis by drug therapy combined with psychological intervention. Ear Nose Throat J. (2020). 10.1177/0145561320902859 32050792

